# A detailed process map for clinical workflow of a new biology‐guided radiotherapy (BgRT) machine

**DOI:** 10.1002/acm2.13606

**Published:** 2022-05-10

**Authors:** Min‐Sig Hwang, Ron Lalonde, M. Saiful Huq

**Affiliations:** ^1^ Division of Medical Physics, Department of Radiation Oncology University of Pittsburgh School of Medicine and UPMC Hillman Cancer Center Pittsburgh Pennsylvania USA; ^2^ Present address: Radiation Oncology Allegheny General Hospital 320 E North Ave Pittsburgh PA 15212 USA

**Keywords:** biology‐guided radiotherapy (BgRT), PET‐Linac, prospective process map, TG100, X1 machine

## Abstract

**Purpose:**

Biology‐guided radiotherapy (BgRT) is a new external beam radiation therapy modality combining PET‐CT with a linear accelerator that has the potential to track and treat one or more tumors in real‐time. The use of PET and radiopharmaceutical tracers introduces new processes that are different from the existing treatment processes. In this study, we have developed a process map for the clinical implementation of a prototype BgRT machine.

**Methods:**

A team of 13 members from various radiation therapy disciplines at our institution participated in developing a prospective process map for a prototype BgRT machine. The methodology provided by the AAPM TG 100 report was followed. In particular, the steps unique to the BgRT workflow, using hypofractionated stereotactic body radiation therapy with fluorodeoxyglucose radiolabeled with fluorine‐18 (FDG) to guide beam delivery, were analyzed.

**Results:**

The multi‐disciplinary team in the department of radiation oncology at our institution developed a prospective process map for the clinical BgRT workflow. By focusing on the appropriate level of detail, 15 major subprocesses, 133 steps, and 248 substeps were identified and the process map was agreed upon as being useful, implementable, and manageable. Seventy‐four steps from nine subprocesses, 55.6% of the whole process, were analyzed to be the BgRT unique steps. They originate mainly from: (1) acquiring multiple PET images at the BgRT machine with separate patient visits, (2) creating a unique biological treatment volume for BgRT plan (PTV_BgRT_), and (3) BgRT plan optimization and treatment delivery using PET images.

**Conclusion:**

Using BgRT to irradiate multiple metastases in the same session will impact clinical workflow, thus a graphical process map depicting the new clinical workflow with an appropriate level of detail is critical for efficient, safe, and high‐quality care. The prospective process map will guide the successful setup and use of the new BgRT system.

## 1 INTRODUCTION

Radiation treatment for multiple metastatic cancers is usually only considered as a palliative modality, where the primary goal is to ease symptoms.^1^, ^2^ However, there is emerging evidence for the clinical benefits of complete metastatic ablation, demonstrating that localized radiation therapy of metastatic tumors is correlated with enhancement of overall survival and progression‐free survival in combination with systemic therapy.^3^, ^4^, ^5^, ^6^ In many different tumor types, the idea of debulking all solid tumors holds great promise for improving outcomes. However, debulking multiple tumors is burdensome, due to the limitations of conventional radiation therapeutic platforms, for example, motion management of the tumor, toxicity to healthy tissue, and long treatment time for the patient.^7^ Hence, there is a need for an innovative technological approach to efficiently and completely ablate multiple metastatic lesions in the same treatment session.

Biology‐guided radiotherapy (BgRT) is a new external beam radiation therapy modality combining PET‐CT with a linear accelerator (PET‐Linac), which utilizes real‐time partial images of radiolabeled tumors to deliver a dynamically tracked dose distribution. The new prototype BgRT enabled system, that is, the Reflexion™ X1 biology‐guided radiotherapy system, has recently been developed by RefleXion Medical, Inc. (Hayward, CA).^6‐8^ When compared to conventional image‐guided radiation therapy, the X1 BgRT system's key technological innovation is to use PET detection of outgoing tumor emissions to localize the tumor and guide the fast‐rotating linac to deliver radiotherapy beamlets with sub‐second latency.^6^, ^7^, ^8^ The direct and continuous feedback loop between the tumor itself and treatment machine allows the system to accurately guide and conform the beamlets to the moving tumor during ongoing treatment.^6^, ^7^, ^8^ Thus, the physiological motion envelope, and all normal tissues within it to ensure the target coverage, can be reduced or removed, that is, avoiding the internal target volume (ITV) approach.^8^ Furthermore, while conventional linacs would need multiple plans to treat multiple metastatic lesions, the combined X1 BgRT system would notably require only a single plan with a single tracer injection to treat the same number of lesions.^6^, ^7^, ^15^

The X1 machine has a ring‐gantry design and consists of six major subsystems. They include a 6MV‐FFF linac, dual 90° arcs of state‐of‐the‐art PET detectors, and a 64‐leaf binary multi‐leaf collimator (MLC) with each leaf transitioning at 100 Hz to shape the beam. The system also houses a kilovoltage (kV) fan‐beam CT scanner for initial patient setup and a megavoltage (MV) detector for optional use in QA. Altogether, these subsystems rotate at 60 RPM on a ring‐gantry platform, delivering radiation from 100 discrete firing positions around the patient.^7^, ^8^ The couch provides an effective 6 degrees‐of‐freedom with roll correction from the gantry.

The combination of radiation therapy and PET in one machine, along with new hardware and advanced algorithms, brings a paradigm change to conventional image‐guided radiation therapy. The use of dual PET detectors and radiotracers for radiation treatment is a departure from current radiation therapy technology and thus will introduce various new processes, fundamentally different from the existing treatment process. According to reports by the World Health Organization (WHO)^9^ and the International Atomic Energy Agency (IAEA),^10^ the rapid adoption of new technologies in a busy clinical setting, together with a lack of clinical staff with experience, and ineffective communication/transfer of essential information are potential contributing factors for incurring errors in the radiation therapy process.^11^, ^12^, ^14^ The pressure to implement this new technology promptly and the lack of highly needed extensive coordination among different groups in the radiation oncology department can create opportunities for incurring errors and delaying the implementation of BgRT. New clinical guidelines are necessary to implement this new technology safely. Therefore, in this study, one of our goals was to first understand how to use the X1 machine for BgRT delivery and then to develop a clinical workflow for the benefit of staff and patients using a prospective process map. In particular, we identified new and unique subprocesses and steps to the clinical BgRT workflow, which could otherwise be abstract for a clinical team with no BgRT experience.

## 2 METHODS AND MATERIALS

The prospective process map that describes the clinical workflow for BgRT using the X1 machine was developed for staff in the department of radiation oncology from the patient's perspective. The process map was designed for an academic radiation oncology department equipped with a PET‐CT simulator with injectable tracers for radiopharmaceutical handling. The PET‐CT protocol already established in clinical use was adapted to accommodate BgRT. The goal of using the X1 machine is to treat multiple metastatic tumors found in various organs including head/neck, lung, liver, and lymph nodes using fluorodeoxyglucose radiolabeled with fluorine‐18 (^18^F‐FDG) as the source for biological guidance for hypofractionated stereotactic body radiation therapy (SBRT). Where appropriate, recommendations from the AAPM TG 100 report were followed.^14^

The new clinical workflow was developed as an extension of the clinical workflow for SBRT that has been in use at our institution for more than a decade. The new workflow is a combination of the existing clinical workflow for SBRT and the subprocesses and steps unique to BgRT. The following BgRT specific unique subprocesses and steps were identified: scheduling of the PET/CT imaging‐only and therapy procedures on the X1 machine, ordering the FDG, performing the FDG injection, processing uptake isolation of the patient post‐injection, transporting the patient to the treatment area from the uptake room, staffing requirements, staff training, licensure and certification needs for handling patients injected with FDG, patient management requirements during‐treatment, processing treatment modifications if PET imaging was unsuccessful, that is, when a usable PET signal is not obtained during the therapy session, and patient management requirements post‐treatment. Finally, substeps and the staff responsible for them were determined.

### 2.1 Development of the graphical clinical BgRT workflow

The process map was developed according to the methodology provided by the AAPM TG 100 report.^14^ Following the recommendations of TG 100 report, a multi‐disciplinary team was formed to develop the process map. As seen in Table 1, the UPMC team consisted of 13 members from various radiation therapy disciplines at our institution: one radiation oncologist, six medical physicists, one medical dosimetrist, two radiation therapists, one nuclear medicine technologist, the radiation safety officer, and one administrative staff. All but one member had at least 10 years of clinical experience in the department of radiation oncology in a busy academic cancer center. One member has seven years of clinical experience in the department. The entire team actively participated in developing a prospective process map for the new X1 machine. One physicist in the team took the lead to call for meetings, to communicate efficiently and effectively, and responsibly consolidate and update the process map. The leading physicist contacted the team members individually as necessary to seek expertise in their particular subspecialty. Further discussions and revisions were conducted until everyone agreed on a final version.

**TABLE 1:** Number of participants by profession involved in developing the new clinical workflow of the BgRT

Profession category	Participants
Radiation oncologist (RO)	1
Medical physicist (MP)	6
Medical dosimetrist	1
Radiation therapist (RT)	2
Nuclear medicine technologist (NMT)	1
Administrative staff (Admin)	1
Radiation safety officer (RSO)	1
Total	13

In August of 2018, there was a kick‐off meeting between the members of the UPMC team and those from RefleXion Medical, where a schematic of the BgRT workflow was discussed and features unique to simulation, plan setup, imaging, plan optimization, and treatment delivery were identified. Then the UPMC team had three scheduled internal meetings to discuss progress, deciding on the key subprocesses and steps in the preliminary clinical workflow. The clinical workflow under development was discussed during the consortium meetings of early X1 machine adopters in 2018 and 2019. Also, meaningful feedback was obtained at two national meetings, AAPM 2019 at San Antonio, TX and the ASTRO 2020 virtual meeting. RefleXion Medical has provided needed feedback about the workflow related to their ongoing development of the prototype machine.

## 3 RESULTS

The multi‐disciplinary team developed a prospective process map for the clinical BgRT workflow which focused on the treatment of multiple metastases in the same treatment session using the X1 machine. By focusing on the appropriate level of detail, 15 major subprocesses, 133 steps, and 248 substeps were identified, and the process map was agreed upon as being useful, implementable, and manageable.

**FIGURE 1:**
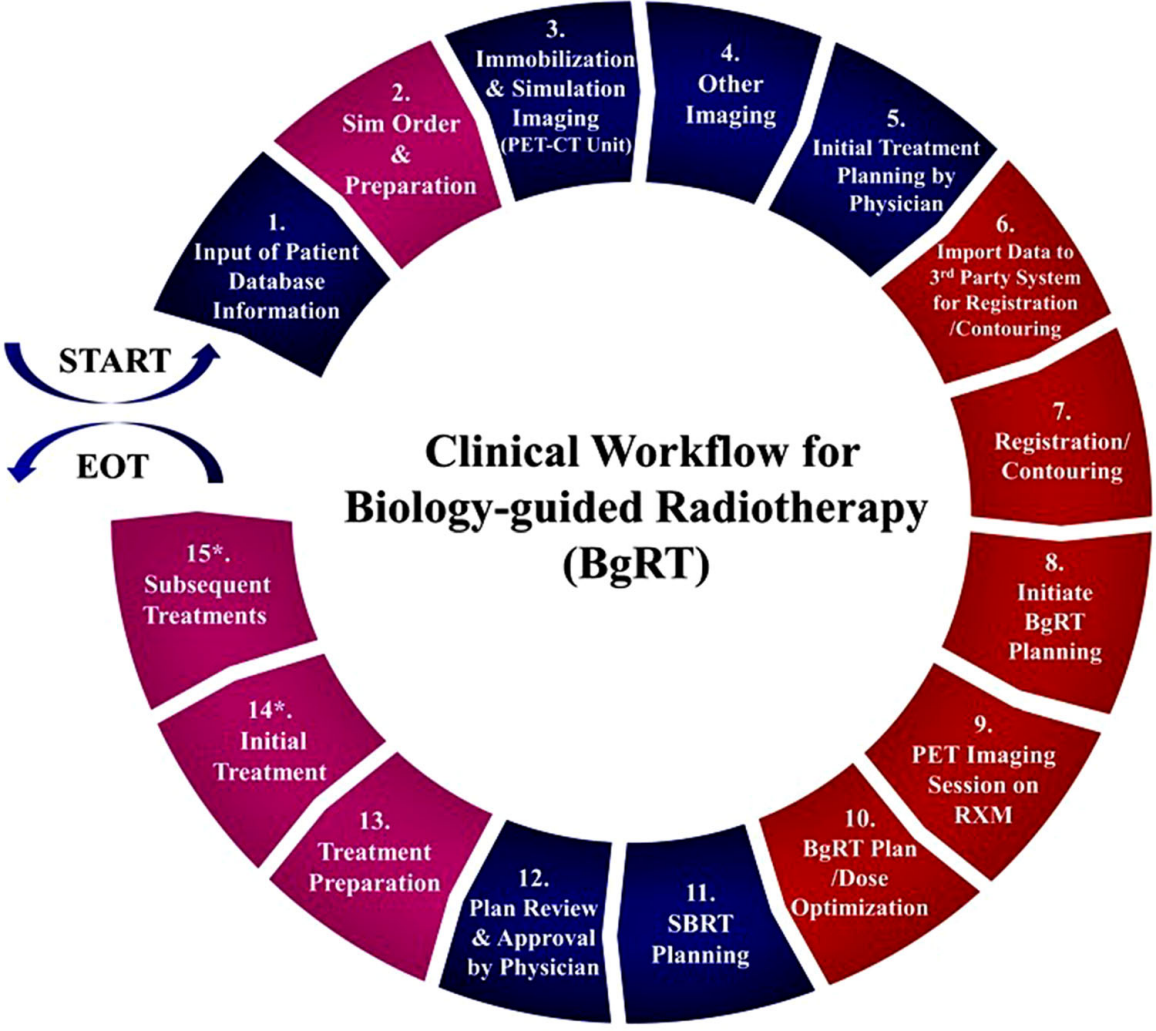
Clinical workflow diagram for biology‐guided radiotherapy (BgRT) using the X1 machine. Segmented blocks in red represent the five new subprocesses (subprocesses 6–10) which hold steps unique to a BgRT workflow. Segmented blocks in magenta (subprocesses 2, 13, 14, and 15) represent four modified subprocesses which hold steps that are unique to a BgRT workflow as well as steps from a conventional SBRT workflow. Segmented blocks in blue (subprocesses 1, 3, 4, 5, 11, and 12) represent the six subprocesses that overlap with a conventional SBRT workflow. * Subprocesses 14 and 15 are divided into two additional sub‐processes; A: PET prescan and go/no go decision, B: subsequent delivery. EOT, end of treatment; RXM, RefleXion biology‐guided radiotherapy machine

Figure 1 shows a graphical representation of the clinical workflow diagram that encompasses the major subprocesses in the BgRT treatment from the time a patient is referred to the department of radiation oncology for BgRT till the end of treatment. As mentioned, the prospective clinical workflow consists of 15 subprocesses. Five subprocesses, shown as segmented blocks in red, are unique to the BgRT process. These are: import data to a 3rd party system for registration and contouring (subprocess 6, i.e., SP6), registration and contouring (SP7), initiate BgRT planning (SP8), PET imaging‐only session on the RefleXion machine (SP9), and BgRT plan and dose optimization (SP10). Segmented blocks in magenta represent four modified subprocesses which hold both steps unique to the BgRT workflow and steps from a conventional SBRT workflow. Those are simulation order and preparation (SP2), initial treatment (SP14), and subsequent treatments (SP15). Six subprocesses, shown as segmented blocks in blue, are part of the standard SBRT process using a conventional linear accelerator.

Figure 2 shows the detailed view of the individual subprocess in the process map. For ease of illustration, the process map is divided into four segments (Figures 2a, 2b, 2c, 2d), each of which focuses on different aspects of the BgRT process in chronological order. Figure 2a focuses on preplanning procedures including the simulation and acquisition of image data sets (SP1–SP5), Figure 2b on the unique BgRT planning procedures (SP6–SP10), Figure 2c on conventional SBRT planning (SP11–SP12) and treatment preparation (SP13), and Figure 2d on the BgRT treatment delivery (SP14–SP15). Notably, the initial treatment and the subsequent treatments are divided into two subprocesses to capture the unique procedures on the BgRT treatment days, for example, using the PET tracer for the radiation treatment; PET prescan and go/no go decision (A, steps 1–10, i.e., S1–10), and subsequent delivery (B, S11–S18). Each individual step of the process map is assigned to a responsible profession group, as shown at the end of each step in the abbreviated form, that is, RO, MP, NMT, RT, and Admin.

**FIGURE 2a:**
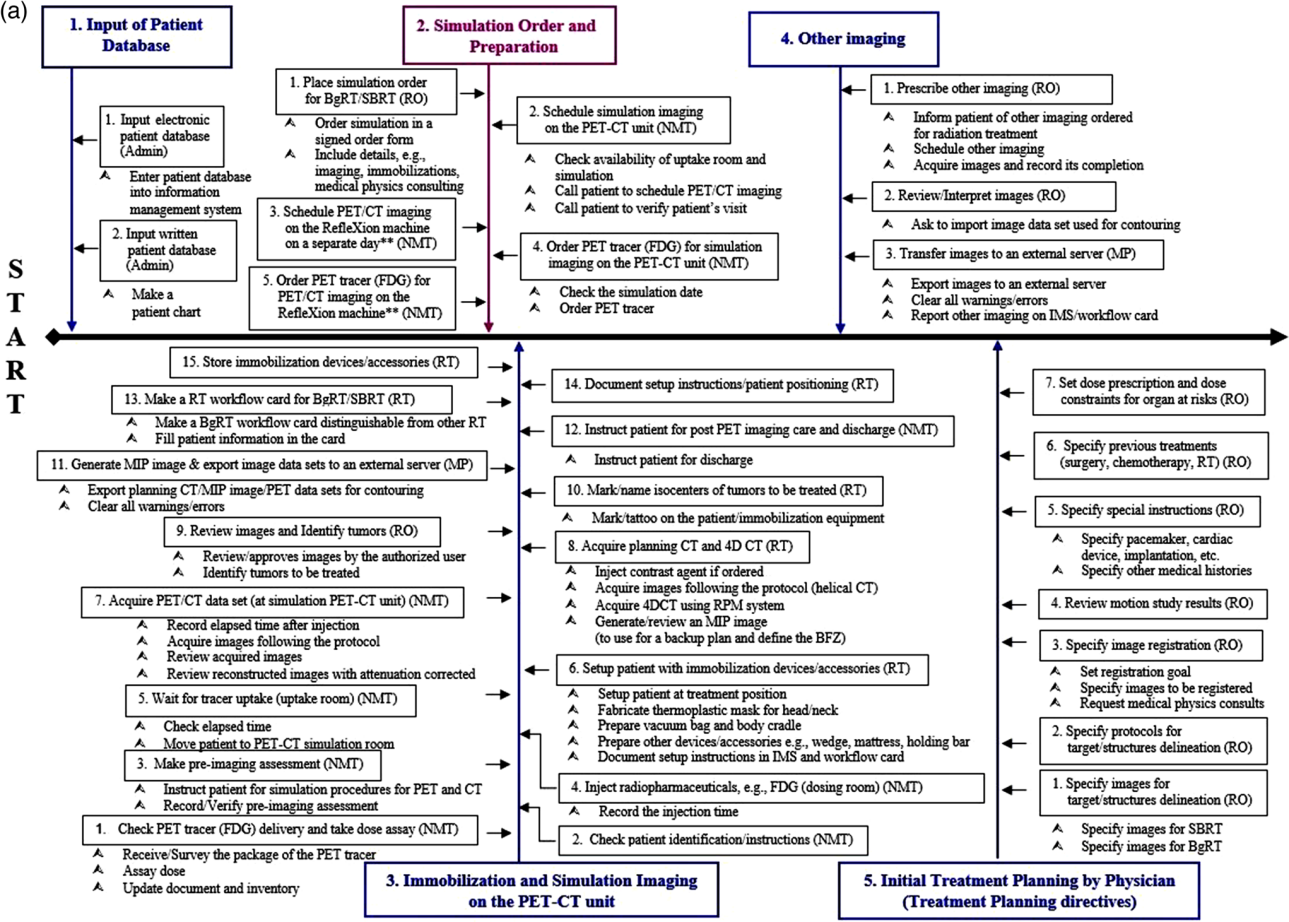
Preplanning procedures including the simulation and acquisition of image data sets (SP1–SP5). Please note the subprocess 2 (SP2) is the modified process, shown in the box in magenta color. **Substeps of steps 3 and 5 in the subprocess 2 are not shown because they are identical to the ones of steps 2 and 4, respectively

**FIGURE 2b:**
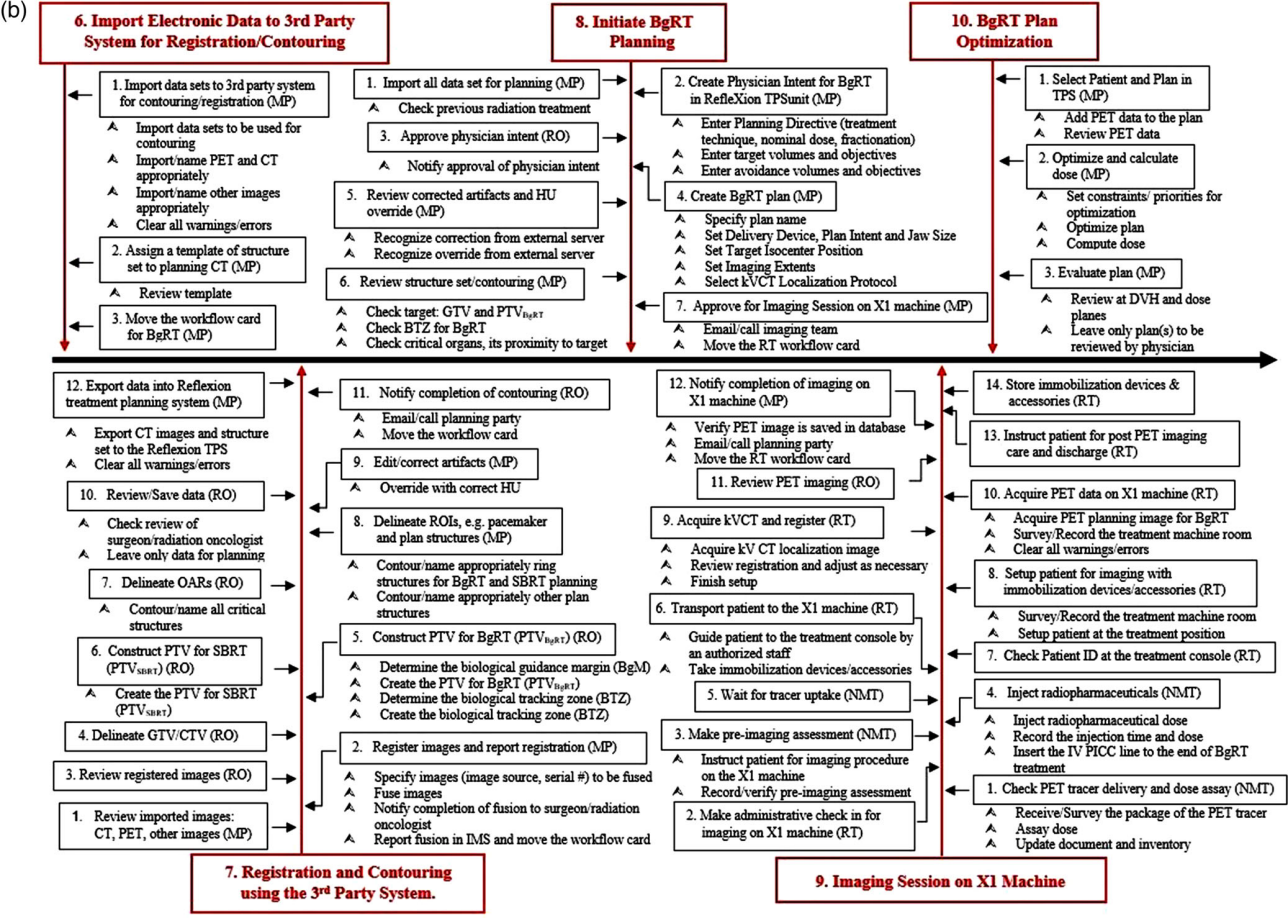
BgRT unique subprocess (SP6–SP10)

**FIGURE 2c:**
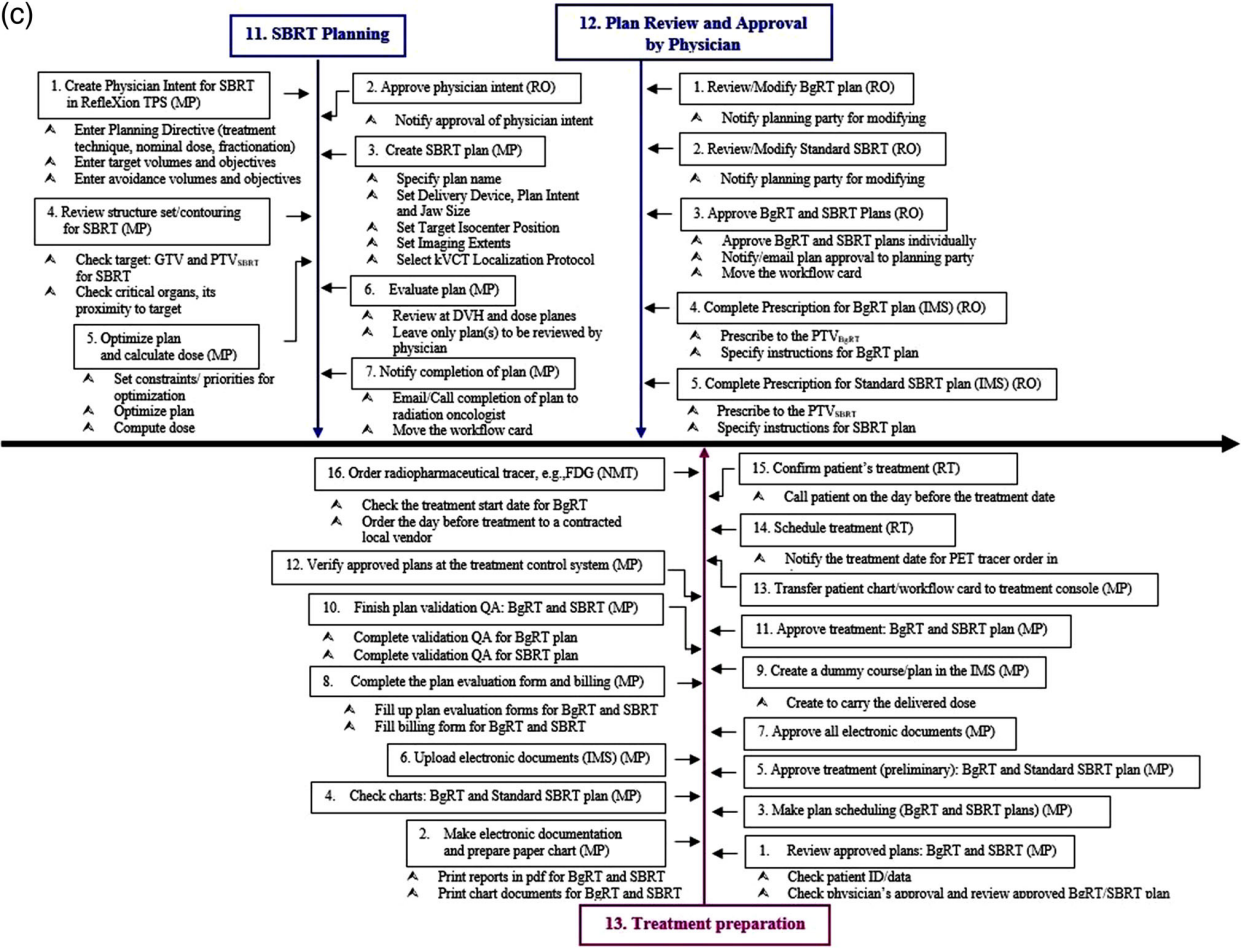
Conventional SBRT planning (SP11–SP12) and treatment preparation (SP13) subprocesses. Please note the subprocess 13 (SP13) is the modified process, shown in the box in magenta color. IMS, information management system

**FIGURE 2d:**
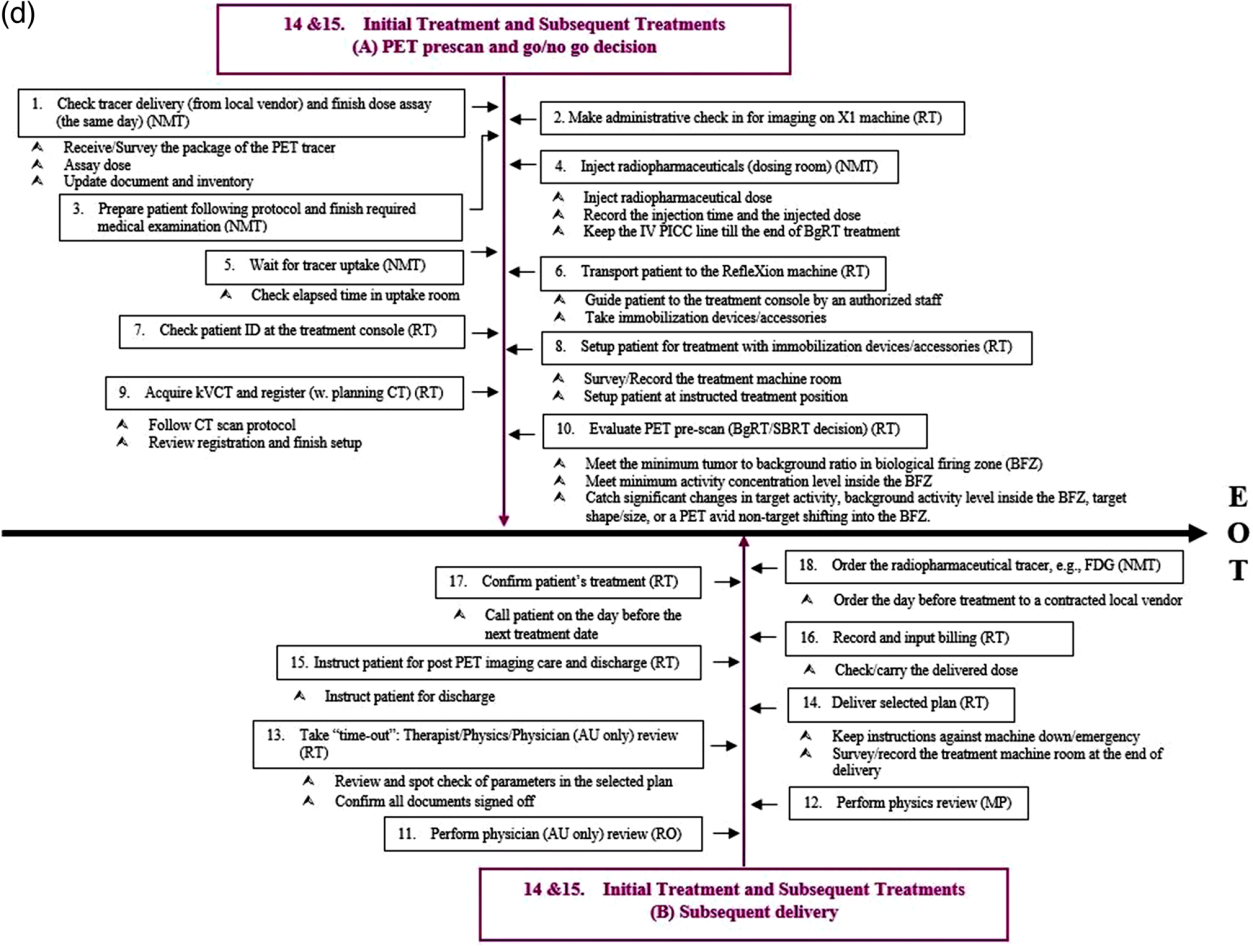
BgRT treatment delivery (SP14–SP15) subprocesses. The BgRT delivery is divided into two subprocesses; A: PET prescan and go/no go decision, B: subsequent delivery. The last fraction will end at step 16. Please note the subprocesses 14 and 15 (SP14 and SP15) are the modified processes, boxes shown in magenta color

Table 2 lists the numbers of steps and substeps identified in the individual subprocesses of the clinical workflow. Out of the total of 133 steps in the whole process, 74 steps (55.6% of the entire steps) are unique to BgRT. Fifty‐nine steps overlapped with steps in the conventional SBRT workflow. Table 2 also includes the numbers of steps tasked to each professional group. Approximately four professional groups took the primary responsibility for the whole process. The medical physicist (MP) group was responsible for most of the steps, namely 41 steps (30.8%). Next, the radiation therapist (RT) group was responsible for 37 steps (27.8%), and the radiation oncologist (RO) group for 28 steps (21.1%). Unlike the conventional SBRT process, but as expected, the nuclear medicine technologist (NMT) was responsible for 25 steps (18.8%). There are two administrative steps for initial patient record and evaluation.

**TABLE 2:** Identified numbers of steps/substeps in the 15 subprocesses of the clinical workflow of Figure 1. Also given are the numbers of steps/substeps that each professional group is assigned. RO, radiation oncologist; MP, medical physicist; NMT, nuclear medicine technologist; RT, radiation therapist. The BgRT unique subprocesses are highlighted in red color, the modified processes in magenta color, and the conventional SBRT subprocesses in blue color

Sub process	Subprocess description	Step/Substep from the whole BgRT process		BgRT unique steps/substeps		No. of steps that each staff is responsible for				
		Step	Substep	Step	Substep	RO	MP	NMT	RT	Admin
1	Input of patient database information	2	2	0	0					2
2	Simulation order and preparation	5	12	2	7	1		4		
3	Immobilization and Simulation imaging on the PET‐CT unit	15	32	0	0	1	1	7	6	
4	Other imaging	3	6	0	0	2	1			
5	Physician treatment planning directives	7	11	0	0	7				
6	Import electronic data to 3rd party system for registration/contouring	3	6	3	6		3			
7	Registration and contouring using the 3rd party system	12	22	12	22	7	5			
8	Initiate BgRT planning	7	17	7	17	1	6			
9	PET imaging‐only session on RXM	14	28	14	28	1	1	4	8	
10	BgRT plan/dose optimization	3	7	3	7		3			
11	SBRT planning	7	18	0	0	1	6			
12	Plan review and approval by physician	5	9	0	0	5				
13	Treatment preparation	16	22	13	19		13	1	2	
14*	Initial treatment	18	29	11	20	1	1	5	11	
15*	Subsequent treatments	16	27	9	18	1	1	4	10	
	Total	133	248	74	146	28	41	25	37	2

## 4 DISCUSSION

To the best of the authors’ knowledge, the work presented in this paper represents the first contribution in the literature describing a clinical workflow for biology‐guided radiotherapy process using the X1 machine. The treatment of multiple metastases in the same session using the ^18^F‐FDG emission tracking is shown to significantly impact the existing SBRT clinical workflow at the authors’ institution; therefore, a graphical process map depicting the new clinical workflow with an appropriate level of detail for simulation, planning, and delivery is critical for efficient, safe, and high‐quality care. For completeness, this effort attempted to capture all possible workflow steps that may occur during the course of BgRT, but not all subprocesses or steps may be required in all cases. For example, other imaging (SP4) may not be required as part of the planning process in many cases. Additionally, this analysis assumed that a stand‐alone PET‐CT unit was used for radiotherapy simulation, which is not strictly required for BgRT planning especially if the patient has received a recent PET‐CT study that can be used for reference. Given these assumptions, we have identified the 74 steps and 146 substeps that are unique to the new BgRT workflow and the relationships between the steps. Four sources of the unique steps in BgRT workflow were identified and are discussed below.

### 4.1 Sources of steps unique to BgRT

#### 4.1.1 Multiple PET imaging sessions

Multiple PET imaging sessions with ^18^F‐FDG play a central role in the BgRT workflow. A patient will receive PET tracer injections multiple times in the course of BgRT treatment planning and delivery: once for acquiring simulation PET‐CT images (SP3), once for acquiring PET images on the X1 machine (SP9), and at every treatment fraction (SP14 and SP15). The first PET imaging with a full axial field of view is acquired at the simulation PET‐CT unit, which is not a part of the X1 machine. In the BgRT workflow, the simulation PET imaging will not be directly used for the BgRT planning and optimization. However, it will be used for two purposes: one is to identify/confirm all metastatic lesions to be treated during the course, in particular, when the tumors are spread throughout the body, small in size, or are difficult to localize in CT (SP7). The other is to contour the target volumes for initializing a BgRT plan, a prerequisite which the new BgRT platform would require to permit PET imaging on the X1 machine (Figure 2b, SP8).

The rest of the PET imaging will be performed on the X1 machine for the PET imaging‐only session (SP9) and each fraction of BgRT delivery (SP14 and SP15). For BgRT planning (SP10) and for evaluating the PET pre‐scan to determine whether to treat the patient using the BgRT plan or the SBRT plan on the treatment day (SP14 and SP15), the X1 PET images are acquired and processed in much the same steps as ones taken for the PET/CT imaging at the simulation PET‐CT unit. During treatment delivery, however, the X1 PET imaging is different from simulation PET imaging. Instead of waiting for the accumulation of a large number of radiation emissions over several minutes, the two 90‐degree arc PET detectors on the X1 machine continuously generate limited‐time sampled (LTS) PET images with a limited axial field of view.^7^, ^13^ This stream of X1 LTS PET “images”, revealing the tumor's biological signature throughout the BgRT delivery, is used to guide BgRT delivery. The X1 PET image data for the real‐time BgRT consists of a much smaller number of emissions acquired in less than a second, for example, measured at 10 times per second. These LTS images are converted into machine instructions that control the radiation beam to deliver the dose specified by the treatment plan with subsecond latency. LTS images continuously signal the tumor locations regardless of tumor motion.^7^, ^12^

Because of the multiple PET imaging, a BgRT patient will have one more visit to the cancer center as compared to the conventional SBRT process, which lengthens the time between the simulation session to the initial treatment (unless the X1 imaging‐only session is done on the same day as the simulation PET‐CT, which would require the user to contour a few select structures in between the two acquisitions). In addition to the typical visits for the radiation therapy, for example, the initial consultation (SP1), the simulation imaging (SP3), and the treatment delivery (SP14 and SP15), the BgRT patient will have another visit for the PET imaging‐only session at X1 machine (SP9). Once the process is well‐established, it is likely to take at least 10 days from the simulation to the first treatment. To have both the simulation PET imaging and the X1 PET imaging only session on the same day may reduce the sim‐to‐treatment time by avoiding the “extra visit,” and result in a lower exposure to the patient and staff with one tracer injection. However, it would be challenging due to timing and having enough PET activity for both the first PET at the simulation unit and the PET imaging‐only session on the X1 machine. Notably, the imaging‐only session on the X1 machine requires a full treatment period on the linac (Figure 2b, SP9). Thus, carrying out both time‐sensitive imaging sessions on the same day would be discouraged.

#### 4.1.2 Two targets and two approved plans in one treatment course

The second source of the BgRT unique steps originates from dealing with two targets and two approved plans in one treatment course (Figure 2a, SP7). Each treatment course will always have two targets: the planning target volume (PTV) for the BgRT, that is, PTV_BgRT_, and the PTV for the standard SBRT, that is, PTV_SBRT_. As defined by the ICRU report #62,^16^ the PTV_SBRT_ is constructed by adding an internal margin to the clinical tumor volume (CTV) to account for tumor motion as well as a setup margin to account for patient setup uncertainties. On the other hand, the PTV_BgRT_ is constructed by adding the biological guidance margin (BgM) to the CTV (S5 in the SP7), with no additional margin for motion. In BgRT, target motion is a much less of a factor because of the rapid response to the PET emissions. The BgM accounts for two unique BgRT features; the potential misalignment of the PET planning data on the X1 machine and the simulation CT, and the residual PET signal tracking error, evaluated by the radiation oncologist from the motion analysis during simulation (Figure 2b, SP7). Without the motion extent contributed to the PTV, the PTV_BgRT_ may have less volume than the PTV_SBRT_. There is another unique structure in the BgRT plan, named the biological tracking zone (BTZ). The BTZ encompasses the target's full range of motion plus the BgM and a setup margin. The BTZ acts as a mask or keyhole which defines the only region where the machine tracks PET signal and is allowed to deliver radiation. During the BgRT delivery, the PTV_BgRT_ will be tracked within the BTZ.^7^, ^15^

As expected, the BgRT planning process is fundamentally different from the conventional SBRT planning process. First, the output of the planning process differs between the two. The output of the SBRT planning process is a set of machine instructions, for example, movement and position of the gantry, the collimator, the couch, and the MLC leaves. In contrast, the output of the BgRT planning process is a set of firing filters that maps the planning PET image to a desired dose distribution to the PTV_BgRT_. The firing filters are calculated as part of optimization during the BgRT planning process.

Second, the BgRT planning process makes choosing the planning CT data set more straightforward. In our institution, the planning CT data set used for SBRT planning is dependent on the treatment site. For example, lung SBRT plans are made on the averaged intensity projection image set generated from the corresponding phase data sets, abdominal SBRT plans on the CT data set of the 50% phase (CT50), and SBRT plans of the head & neck area and the pelvic area on the helical CT. However, only the helical CT will be used for planning and localization on the BgRT platform.

Third, the respiratory motion management during delivery of the conventional SBRT plan depends on the selected techniques, for example, gated treatment with amplitude or phase gating, deep inhalation breath‐hold, active breathing control, abdominal compression, and triggered real‐time monitoring, but BgRT delivery is simplified, not requiring the motion management during the delivery because the tumor motion is seen as relatively static to the gantry rotating at 60 RPM on a ring platform.

In the workflow, the standard SBRT plan for the PTV_SBRT_ will be ready as a non‐inferior backup plan against when a usable PET signal is not obtained during the treatment session (Figure 2d, S10 in SP14 and SP15). Consequently, two ready‐to‐go plans, the BgRT plan and the SBRT plan, made for the different targets in size and shape will always be seen on the BgRT platform until the course of treatment ends. This will require the therapist to select the right plan to deliver on every fraction. The plan validation QAs of both plans and their documentation will also be a prerequisite to treatment delivery; thus, we will need to consider the time to finish the QA task. Since the daily plan delivered will be the same throughout the course with the daily PET being used only for targeting, one plan validation QA will be necessary (S10 in SP13). In addition, in case the patient ends up with mixed treatments of the BgRT and the SBRT plans, the final doses delivered to the selected PTV or GTV or the organs at risk (OARs) will need to be reconstructed to determine and record in the designated information management system. The BgRT plan QA will not only confirm the ability of the linac to accurately deliver a specific plan and catch upstream machine errors, but also include PET evaluation. Besides the plan validation QAs, the machine‐specific QAs for BgRT should be performed. In particular, measures to ensure consistent X1 PET image qualities and accurate response of the fast‐rotating linac to deliver radiotherapy beamlets with sub‐second latency should be tested routinely.

#### 4.1.3 The BgRT treatment delivery

As mentioned in the results section, the initial treatment (Figure 2d, 18 steps, SP14) and the subsequent treatments (Figure 2d, 16 steps, SP15) are divided into two sub‐processes; (A) PET prescan and go/no go decision (S1–S10), and (B) subsequent delivery (S11–S18(6)). On the treatment day, a BgRT patient goes through the institutional PET preparation procedures: an FDG injection, the standard uptake period, and moving to the X1 machine. After the kVCT localizes the patient to the planning CT, a quick PET prescan is acquired. Immediately, the preconfigured algorithm will evaluate the PET prescan images as to whether or not it meets the minimum tumor to background ratio in the BTZ and the minimum activity concentration level inside the BTZ. The system also performs a dose calculation using the prescan image and verifies that the dose predicted from the prescan image is consistent with the approved BgRT plan. If there are any significant changes in target activity, background activity level inside the BTZ, changes to target shape/size, or a PET avid non‐target shifting into the BTZ that prevent the predicted dose from agreeing with the approved plan, the system will not proceed with BgRT delivery. Once the algorithm makes the "go" decision (S10 in SP14 and SP15), then the BgRT plan is ready to be delivered with real‐time feedback between the PET detectors and the linac within the fast rotating BgRT platform.

#### 4.1.4 Using a 3rd party system for registration/contouring

The workflow needs to use an external treatment planning system (Figure 2b, SP6 and SP7) as well as the RefleXion BgRT platform. The 3rd party system will be required to register the diagnostic images to the planning CT and contour the target volume and organs at risk. Also, the examination of image artifacts and their correction, for example, Hounsfield unit (HU) correction, will be made in the 3rd party system (S5 in SP8).

### 4.2 Key components for the successful implementation

There are seventy‐four BgRT unique steps that are not part of the workflow of conventional SBRT. These unique steps may be sources of potential failure. It would be key to the successful implementation of the new clinical workflow that each professional group builds up a solid understanding of the details of the unique steps and their place in the overall process. The key components to be considered are management of the time‐sensitive PET tracers for the BgRT, personnel considerations for the BgRT program, and preparedness for emergencies.

#### 4.2.1 Management of the time‐sensitive PET tracers for the BgRT

The subprocesses on the X1 machine, the imaging‐only session (Figure 2b, SP8) and the treatment delivery (Figure 2d, SP14 and SP15), begin with the PET tracer's delivery followed by the dose assay, and end with the order of the tracer for the next use. Thus, it is important to timely manage the PET tracer as planned. As directed by the current institutional PET‐CT protocol, the tracer delivery, package survey, and dose assay are the first tasks for the nuclear medicine technologist to do on the days that the PET tracer injection is planned. The injection room, the tracer uptake room, and the hot lab are used as designated in the current protocol. The nuclear medicine technologist is in charge of the tasks related to the injected patient care and the transportation agent for delivering the right dose on time. Routine communication and cross‐check between the nuclear medicine technologist and the radiation therapist, and scheduling/confirming the BgRT treatment with the patient, are the keys to seamless operation. In particular, when multiple BgRT patients are scheduled for PET imaging at the X1 machine and the simulation PET‐CT unit, vigilant cross‐check of the patient's scheduling/check‐in status is always emphasized. It is indispensable to keep strong cooperation with the local tracer supply vendor.

#### 4.2.2 Personnel considerations for the BgRT program

The complexity and lack of experience in BgRT will require an increased level of staff involvement in every aspect of the BgRT process. During the initial clinical implementation of the new workflow using the X1 machine, qualified radiation oncology staff should form the clinical team to implement the BgRT workflow. The minimum staffing requirement for each profession, for example, radiation oncologist, medical physicist, nuclear medicine technologist, and radiation therapist, and maximally allowed daily BgRT patients for the imaging‐only session and treatment delivery should be derived in advance.^16^, ^17^ Additional staff resources will be needed to maintain a high‐quality BgRT program.

To the best of our knowledge, there is currently no federal regulation about the authorized user for the use of unsealed byproduct materials for radiation treatment. Hence, the regulations for imaging and localization studies^18^ might be applied to the authorized user of PET tracers for the BgRT treatment machine. The regulations require specific board certification, work experience, and training under supervision for the radiation oncologist to be the authorized user for BgRT using FDG. Therefore, it is advised that any radiation oncologist wishing to be the authorized user for BgRT finishes training/education in advance, unless a qualified nuclear medicine physician is available at the X1 machine console room for supervising the FDG tracer use and analyzing PET data.

#### 4.2.3 Preparedness for emergencies

Besides typical emergencies during radiation therapy, the use of the PET tracer for the BgRT may bring the possibility of new emergencies, for example, the machine down during the BgRT delivery of the radiolabeled patient. Detailed safety check procedures in the BgRT treatment room, caring for the radiolabeled patient, minimizing the exposure to the staff and other patients, obtaining prompt field service, etc., must be developed, along with appropriate training.

#### 4.2.4 Limitations of the current BgRT process map

As mentioned in the previous section, the process map targets being comprehensive for implementing clinical BgRT workflow and is originally framed based on the process tree of the AAPM TG 100 report. Some superfluous details and substeps may be included in the process map, which may detract from its purpose of highlighting the unique elements of BgRT, and some subprocesses may be integrated into other subprocesses (i.e., Subprocess 1: Input of Patient Database is common to any patient and can be combined into subprocess 2).

The new process map has also been developed for PET‐CT equipped academic centers. Thus, it will need modification if a stand‐alone center or a satellite hospital is planning to install the X1 machine. In these settings, patient transportation between the injection suite and the X1 machine may be a new key issue to BgRT implementation, and since BgRT is projected to use SBRT fractionation, usually having less than five fractions, longer fractionation schemes are not considered in the BgRT workflow.

## 5 SUMMARY

This paper describes for the first time a prospective clinical workflow for the implementation of BgRT using the new X1 machine. As expected, the treatment of multiple metastases in the same session with FDG‐based image guidance will impact clinical workflow. Therefore, a graphical process map depicting the new clinical workflow with an appropriate level of detail for preplanning, planning, and delivery is critical for efficient, safe, and high‐quality care. We have identified 74 steps that are unique to this new clinical process, as well as the relationships between the steps, and have highlighted the key components to be considered for the successful implementation of the clinical workflow. The minimum staffing requirement for each profession, (e.g., RO, MP, NMT, and RT), and maximally allowed daily BgRT patients must be determined. Staff training/education to meet regulatory requirements, in particular for the RO as an authorized tracer user, needs to be considered. Strong cooperation with a local PET tracer vendor and creating patient schedules to allow for multiple time‐sensitive PET images at the treatment machine are also keys to the operation of the new BgRT workflow.

At the time of this writing, the X1 machine has received 510(k) clearance from the United States Food and Drug Administration (FDA) for delivering SBRT, stereotactic radiosurgery (SRS), and intensity‐modulated radiotherapy (IMRT).^19^ BgRT is awaiting FDA clearance. Thus, the prospective process map along with a detailed written description will serve as useful instructions to guide the successful clinical implementation of the BgRT treatment using the new BgRT machine when the BgRT capability of the X1 machine becomes approved for clinical use. Finally, the process map can be used as a framework for failure mode and effect analysis. The subprocesses, steps, and substeps unique to BgRT represent sources for many potential failure modes in the new BgRT process. High‐risk steps identified by performing a prospective risk analysis will be used to instruct the new therapeutic strategy and to develop a risk‐based quality management program for BgRT.

## CONFLICT OF INTEREST

The authors have no conflict of interest.

## AUTHOR CONTRIBUTIONS

All authors substantially contributed to the conception or design of the new clinical process map for the BgRT and analyzed/interpreted results. Also, all authors contributed to drafting the manuscript or revising it critically for important intellectual content.

## Supporting information

Supporting Information

## Data Availability

The data used to support the findings of this study are included within this article.
