# Supplementary material for: A detailed process map for clinical workflow of a new biology‐guided radiotherapy (BgRT) machine
Source: J Appl Clin Med Phys. 2022 May 10;23(6):e13606. doi: 10.1002/acm2.13606 (PMC9194983; doi:10.1002/acm2.13606)
Supplement: Supplementary file 1 — Supporting Information [file ACM2-23-e13606-s001.docx]

**A Detailed Process Map for Clinical Workflow of a New Biology-guided Radiotherapy (BgRT) Machine**

Min-Sig Hwang*, Ron Lalonde, and M. Saiful Huq

Division of Medical Physics

Department of Radiation Oncology

University of Pittsburgh School of Medicine and UPMC Hillman Cancer Center, Pittsburgh, PA

*Corresponding author: Min-Sig Hwang, min-sig.hwang@ahn.org

*Present address: Radiation Oncology, Allegheny General Hospital, 320 E North Ave, Pittsburgh, PA 15212

Suggested Running Title: A Detailed Process Map for Clinical Workflow of a New Biology-guided Radiotherapy (BgRT) Machine

Author Contribution Statement: All authors substantially contributed to the conception or design of the new clinical process map for the BgRT and analyzed/interpreted results. Also all authors contributed to drafting the manuscript or revising it critically for important intellectual content.

**Acknowledgements**

This research was supported in part by RefleXion Medical (Hayward, CA). The authors would like to thank Angela Da Silva, Ann Yang, Kathy O’Shaughnessy, Pia Kaur, Samuel Marzin, Sean Shirvani, and Thomas Cornwell for providing valuable comments. Min-Sig Hwang would specially like to express his thanks and sincere gratitude to Dr. Si Young Jang for many fruitful discussions and insightful comments on building up the prospective BgRT process map.
